# Association of Piriformis Thickness, Hip Muscle Strength, and Low Back Pain Patients with and without Piriformis Syndrome in Malaysia

**DOI:** 10.3390/life13051208

**Published:** 2023-05-18

**Authors:** Ida Kartini Othman, Naresh Bhaskar Raj, Chua Siew Kuan, Sabrilhakim Sidek, Ling Shing Wong, Sinouvassane Djearamane, Annaletchumy Loganathan, Siddharthan Selvaraj

**Affiliations:** 1 Centre of Physiotherapy, Faculty of Health Sciences, Universiti Teknologi MARA, Puncak Alam 42300, Malaysia; chuasiewkuan@gmail.com (C.S.K.); sabrilhakim@salam.uitm.edu.my (S.S.); 2 Physiotherapy Unit, Hospital Sultanah Nur Zahirah, Jalan Sultan Mahmud, Kuala Terengganu 20400, Malaysia; 3 School of Rehabilitation Science, Faculty of Health Sciences, University Sultan Zainal Abidin (UniSZA), Kuala Nerus 21300, Malaysia; bnaresh@unisza.edu.my; 4 Faculty of Applied Science, Lincoln University College, Petaling Jaya 47301, Malaysia; 5 Department of Radiology, Fiona Stanley Hospital, 11 Robin Warren Drive, Murdoch, WA 6150, Australia; 6 Faculty of Health and Life Sciences, INTI International University, Nilai 71800, Malaysia; 7 Biomedical Research Unit and Lab Animal Research Centre, Saveetha Dental College, Saveetha Institute of Medical and Technical Sciences, Saveetha University, Chennai 602105, India; drsino31@gmail.com; 8 Department of Allied Health Sciences, Faculty of Science, Universiti Tunku Abdul Rahman, Jalan Universiti, Bandar Barat, Kampar 31900, Malaysia; 9 Faculty of Dentistry, AIMST University, Bedong 08100, Malaysia; sidzcristiano@gmail.com

**Keywords:** piriformis syndrome, piriformis thickness, gluteus muscles, muscle activation, low back pain, human health

## Abstract

Low back pain is a serious threat to human health and the illness jeopardizes the human workforce and pressurizes the health system in the community. Low back pain might be related to piriformis syndrome (PS), which is a disorder presented as muscular spasm and hypertrophy that is strongly associated with piriformis thickness. Nevertheless, the relationship between piriformis thickness and morphological and functional changes of the gluteal muscles in PS remains unclear. This study aimed to investigate the association between the thickness, strength, and activation of piriformis and gluteus muscles (maximus and medius) among low back pain (LBP) patients with and without PS. This is a case-control study conducted at HSNZ and UiTM from 2019–2020. A total number of 91 participants (LBP + PS (*n* = 36), LBP − PS (*n* = 24), and healthy (*n* = 31)) were recruited in this study. Negative radiography, specific symptoms, and a positive PS test were applied for PS diagnoses. The thickness, strength, and activation of piriformis and gluteus muscles were measured using ultrasonography (USG) and a surface electromyogram, respectively. Resultantly, the one-way ANOVA test demonstrated no significant difference in piriformis thickness between LBP + PS and LBP − PS (*p* > 0.01). Piriformis thickness was inversely correlated with gluteus maximus strength (r = −0.4, *p* < 0.05) and positively correlated with gluteus medius activation (r = 0.48, *p* < 0.01) in LBP + PS. Stepwise linear regression for LBP + PS revealed a significant association between piriformis thickness and gluteus maximus strength (R = −0.34, accounted for 11% of the variance) and gluteus medius activation in prone lying with the hip in an externally rotated, abducted, and extended (ERABEX) position (R = 0.43, accounted for 23% of the variance). With the adjustment of age and gender, piriformis thickness, gluteus maximus strength, and gluteus medius activation in prone lying with hip ERABEX demonstrated a significant association, but no independent effect of age and gender was detected within the range. Meanwhile, a significant association between piriformis thickness and gluteus maximus thickness was observed (R = 0.44, accounted for 19% of the variance) in the LBP − PS group. These findings may assist to elucidate the actions and functions of piriformis and gluteus muscle in LBP with and without PS.

## 1. Introduction

Low back pain (LBP) is the pain generated between the lower 12th rib margins and the lower gluteal folds [1]. LBP is among the leading causes of disability worldwide [2], with 37% of patients reporting at least monthly experiences or recurrent episodes of back pain [3]. Similar results were observed in previous surveys conducted in Malaysia, as the incidence of LBP was approximately 12% in a semi-rural community [4], whereas the prevalence of LBP among commercial vehicle drivers and medical students was high at 60% and 68%, respectively [4,5].

Patients with LBP might also conjunctionally suffer from piriformis syndrome (PS). PS is a complex condition that is often underdiagnosed or misdiagnosed as chronic hip pathologies and LBP [6], with the prevalence ranging widely from 0.3% to 36% [6,7]. In Malaysia, approximately 17.2% of patients with LBP and chronic buttock pain were clinically diagnosed with PS [7]. Furthermore, PS is among the primary causes of sciatica, buttock pain, and LBP [7,8].

Piriformis syndrome (PS) is a multifactorial disorder with age, gender, and work-related factors contributing significantly to the incidence in high-risk populations. Most researchers identified the presence of PS as deep gluteal or buttock pain [9,10] while a few studies described it as non-discogenic sciatica, indirectly implying non-specific LBP [7,11]. The diagnostic criteria vary due to the wide range of the suspected aetiology of PS [7].

Patients with non-specific LBP commonly experience instability in the spinal and pelvic-hip column. Evidence from the literature reflects the interconnection between the piriformis and hip muscles in providing stability and control of the lumbopelvic-hip complex [12]. Back muscle control and the stability of the lumbopelvic region are important to transfer forces between the spine and lower limbs [13] at the thoracolumbar fascia when performing multitask activities [14]. The three muscles: gluteus maximus, piriformis, and posterior fibre of the gluteus medius, are recognised as sustaining trunk and pelvic rotational activities while bearing weight over one limb [12]. Piriformis partly provides a major component of lumbopelvic and hip stability and acts as a short hip external rotator and short hip abductor [12]. In addition, Giphart et al. (2012) [15] documented that the maximum piriformis activation occurs when performing static hip external rotation and abduction with a slight extension or when in the prone lying position. Considering these two positions are the best possible functional activities for piriformis and gluteus muscle actions, the current study concentrates on visualising the muscle activation of the hip extensor and rotator muscle relative to piriformis thickness.

Piriformis tightness, a spasm following overloading or overuse, has been determined as the major cause of LBP. PS is completely clinical and highly related to the abnormal condition of the piriformis muscle, which is usually traumatic in origin. Thus, hypertrophy or an increase in the vertical thickness of the piriformis fibre has been considered one of the clinical symptoms of PS.

While the piriformis and hip rotator muscles are known to provide relative dynamic stability and control to the lumbopelvic-hip region [12], few studies have highlighted the relationship between PS with alterations in the piriformis and the morphology or functions of the hip muscles. The data paucity might be due to the deep anatomical locations of the piriformis muscle or an ongoing debate regarding the diagnosis and management of PS [16]. Likewise, malfunction of the piriformis-gluteus interactions triggers further impairment in the presence of hypertrophic piriformis—which is the most recognised form in PS. Nonetheless, information on the relationship between the piriformis and hip muscle morphology and function remains scarce and uncertain. Conclusively, no study has been undertaken to address the relationship between the piriformis and gluteus muscles in LBP or PS.

Therefore, this study aims to investigate the association between piriformis muscle thickness (depicted as hypertrophic) and the gluteus muscle functions in LBP with and without PS. The specific objectives of this study were to (1) to compare the piriformis thickness in LBP patients with and without PS, (2) to compare the gluteus maximus and gluteus medius thickness, strength, and activation in two different functional positions: prone lying and single-leg standing (SLS), (3) to determine the relationship between the piriformis and gluteus maximus and gluteus medius thickness, strength, and activation, and (4) to determine effects of age and gender in the aforementioned relationships in LBP patients with and without PS.

## 2. Materials and Methods

### 2.1. Study Design, Population, and Location

This study is a case-control research design. The study recruited adults aged between 20 and 60 years and diagnosed with non-specific LBP from the Physiotherapy Clinic, Hospital Sultanah Nur Zahirah (HSNZ), Kuala Terengganu and the Physiotherapy Clinic, Universiti Teknologi MARA (UiTM), Puncak Alam. Both clinics were selected based on the availability of an ultrasonography machine and LBP cases for data collection. One site investigator, an orthopaedic surgeon, was also appointed from HSNZ to offer direct consultation to patients throughout the data collection process.

For the control group, participants were recruited among patients attending the clinic or clinic staff who met the inclusion criteria. Data collection, including the interviews, preliminary examinations, piriformis specific tests, and electromyogram (EMG) tests, was conducted in the treatment rooms at the physiotherapy clinics. Meanwhile, the USG procedure was performed in the Pain Management Room, Anaesthesiology Department, HSNZ and Medical Imaging Lab, UiTM. The data collection was conducted from September 2019 to June 2020.

### 2.2. Sampling Method

A simple randomised sampling was applied for both the LBP and healthy groups. Patients registered at the clinics from July 2019 to June 2020 were considered for sampling. A list of new cases of patients diagnosed with LBP and referred to the Physiotherapy Clinic was granted from the Hospital Information System (HIS) by HSNZ, whereas an appointment listing book was provided by the UiTM Physiotherapy Clinic. All the patients were numbered and had an equal probability of being chosen to participate. Participants were randomly selected based on the numbered name list and screened for eligibility. Only those who met the inclusion criteria were given an appointment call.

### 2.3. Inclusion and Exclusion Criteria

All the patients diagnosed with non-specific LBP by orthopaedists were randomly enrolled and screened for inclusion and exclusion criteria. For LBP + PS and LBP − PS groups, patients were included if aged 20 to 60 years old, of both genders, with body mass index (BMI) ranging from 20 to 30 kg/m^2^, presented with or without radiculopathy, mild pain (1–3 on VAS), and normal radiography imaging. Meanwhile, patients with any history of back or lower limb surgery, acute LBP, or those who had experienced LBP within the last six months, diagnosed cases of rheumatoid arthritis or osteoarthritis of the spine or hips, those with a spinal deformity or neurological disease, pregnant females, physically active individuals (two or more strenuous activity sessions per week), those undertaking an active exercise programme for back, buttock, or hip pain at present, and those who were uncooperative, were excluded. Participants who fulfilled the criteria and demonstrated positive symptoms in the subjective and objective examination of PS were grouped into the LBP + PS group, whereas those who did not show positive findings in the subjective and objective examination, especially specific piriformis tests, were grouped into the LBP − PS group.

Those who were not diagnosed with LBP; with no back or hip pain symptoms within the previous six months, no history of trauma in the spine or lower extremities, and no history of back surgery or lower limb surgery; pregnant women; physically active individuals (two or more strenuous activity sessions per week) and those not attending any ongoing back exercise programme were enrolled as healthy participants.

### 2.4. Sample Size Calculation

Each of the research objectives was considered for the sample size calculation while utilising Green’s formula. A standard deviation (SD) of 3.66 was used as the reference [17] to compare the piriformis thickness among LBP + PS, LBP − PS, and healthy adults. A minimum sample size of 33 was obtained upon using Power and Sample Size Calculation Software version 3.1.2. To compare the gluteus maximus and gluteus medius muscle strength between the three groups, an SD value of 25.0 maximal voluntary contractions (MVC1) and 32.0 (MVC2) was referred to [18]. Assuming these parameters and using the Power and Sample Size Calculation Software resulted in the same sample size of 33 for each group. For the third objective, which is to determine the relationship between piriformis thickness and hip muscle strength and activation in the study groups, an SD value of 0.36 [19] was referred to. Using Green’s formula (1991) with a power of 0.80 and effect size of 0.05, the sample size calculated for each group was 24. Based on these calculations, a minimum of 33 participants was targeted for each group. Considering a 10% dropout tendency, the final sample size model was 36 for the experimental group, while the total number of participants was 91.

### 2.5. Ethics Approval

Ethical approvals were obtained from authorised institutional research committees, involving the Medical Research Ethical Committee (MREC) (NMRR-19-1302-45661 (IIR), the Ministry of Health Malaysia) and the Research Ethical Committee (REC) (600-IRMI (5/1/6), Universiti Teknologi MARA (UiTM). All the participants were briefed regarding the study procedures prior to participation, while verbal consent to participate was granted via phone calls. Written informed consent was obtained voluntarily before commencing the study and participants were allowed to withdraw at any time at their request.

### 2.6. Instrument and Outcome Measure

#### 2.6.1. Anthropometry

The participants’ heights were measured using a portable stadiometer (Seca^®^ 284 Stadiometer, Seca, Birmingham, UK) and recorded to the nearest 0.1 cm. The participants stood with their heels, buttock and scapulae in contact with the stadiometer. A researcher verified that the participant maintained the maximum erect posture after positioning and that the participant’s head was in the Frankfort Horizontal Plane. Bodyweight (kg), BMI (kg/m^2^), muscle mass composition (%), and fat mass composition (%) were determined using a digital body fat analyser machine and recorded to the nearest 0.1 kg. Participants were required to stand erect on the platform and grasp their hands firmly. During measurement, the body should remain still with a 90-degree shoulder flexion. All the data were calculated automatically after the whole-body scan had been completed.

#### 2.6.2. Identification of Piriformis

Abnormality of the piriformis was identified through palpation over the muscle belly of the ipsilateral leg [20]. The patients were positioned in a side-lying pose, with the affected leg uppermost with the hip and knee flexed to 90 degrees for easy palpation. After the palpation in side-lying position, the participants were held lying prone on the examination table. Prior to palpation, an imaginary line was drawn immediately from the greater trochanter to the cephalic border of the greater sciatic foramen at the sacrum, and the line was divided into equal thirds. Participants responded positively if there was increased pain at the palpated site. In spasm conditions, an examiner might palpate the sausage-shaped mass in the buttock due to piriformis muscle contraction [21].

#### 2.6.3. Screening for Piriformis Syndrome

Diagnosis of PS was confirmed by positive responses to piriformis specific tests, involving the Lasègue sign, the flexion, adduction, internal rotation (FAIR) test, the heel contralateral knee (HCLK) test, the Active Piriformis test, and the straight leg raise (SLR) test [20]. The FAIR test is a piriformis tension test in which the hip is flexed, adducted, and internally rotated [20]. The FAIR test was conducted by positioning the patient on the examination table on their side, lying with the hip and knee flexed to 60° and 90°, accordingly. While stabilising the hip, the examiner internally rotated and adducted the hip by applying downward pressure to the knee. A positive sign was documented when the procedure resulted in the exacerbation of pain in the area [22].

The Lasègue sign was performed with each participant lying supine on the examination table and the dominant hip passively flexed to 90°. The examiner’s thumb palpated on the point of maximum trigger-point tenderness at the greater sciatic notch, just lateral to the junction of the middle and last third of the line [23]. This provokes pain, indicating a positive sign of PS. HCLK was another test performed to evaluate the involvement of hip joint pathologies. Participants were positioned lying supine on the examination table. The ipsilateral hip and knee were flexed onto the contralateral knee and compression was applied towards it. The Active Piriformis test was performed afterwards to provoke pain in the piriformis muscle, which placed it forcefully in a lengthened position. Any active inflammation or spasm of the piriformis muscle triggered the pain, which indicated a positive sign. For sensitivity, the same examiner, who is the main researcher (IKO), performed the test on all the participants. Participants with positive signs from both diagnostic tests were designated as Group I, while those who showed no sign in one or both tests were designated as Groups II and III, respectively.

### 2.7. Ultrasonography Examination

#### 2.7.1. Intraclass Correlation Coefficient (ICC)

Intraclass correlation coefficients (ICC) were obtained before actual data collection. A group of participants was recruited for a pilot study for each ICC estimation (*n* = 10). The intrarater reliability method was performed for the USG and sEMG applications, whereas interrater reliability was ascertained for manual muscle resistance interrater reliability. ICC values were estimated at 95% confidence intervals and were classified for reliability as described by Koo and Li (2016) [24].

#### 2.7.2. Intraclass Correlation Coefficient of USG

Intraclass correlation coefficients (ICC) were obtained by employing the intrarater reliability method with the USG application for measuring muscle thickness. Based on single measurements, the consistency, and the two-way mixed-effects model, a high degree of reliability was identified between the USG measurements of muscle thickness (ICC = 0.99, 95% CI 0.97–0.99).

#### 2.7.3. Ultrasonography Measurement of Muscle Thickness

Muscle thickness was measured by ultrasonography (USG) (Mindray Ultrasound System DC30, transducer: CH 5-2, Mindray Bio-Medical Electronics Co., Dusseldorf, Germany). The procedure was performed by the main examiner (IKO), a senior physiotherapist who had received special training in musculoskeletal ultrasonography conducted in Universiti Malaya. IKO has also undergone 25 h of live training and demonstration under a musculoskeletal radiologist and radiographer. Prior to application, a standardised USG protocol for measuring piriformis, gluteus maximus, and gluteus medius thickness was developed. A pilot study was conducted to evaluate the ICC. For quality assurance measurement, the USG unit was calibrated weekly by trained staff following manufacturer recommendations.

#### 2.7.4. Piriformis Thickness

Each participant was instructed to lie on the examination table in the FAIR position with the gluteal area exposed [25]. An imaginary line was drawn to allocate the piriformis muscle by finding the intersection of a line extending from the greater trochanter to the ipsilateral posterior superior iliac spine (PSIS) and a second line extending from the ischial tuberosity and the ipsilateral anterior superior iliac spine (ASIS) [19,26]. Aquasonic clear ultrasound gel was placed over the targeted area as a conductive medium. The participant’s details were entered into the internal device and the pre-set hip parameters with the B-mode programme were selected. A curvilinear probe (35C50P) with a transducer, adjusted from 3.5 MHz to 5 MHz, was applied in a longitudinal plane in the area of the piriformis muscle, guided by the imaginary line [19,27].

**Figure 1:**
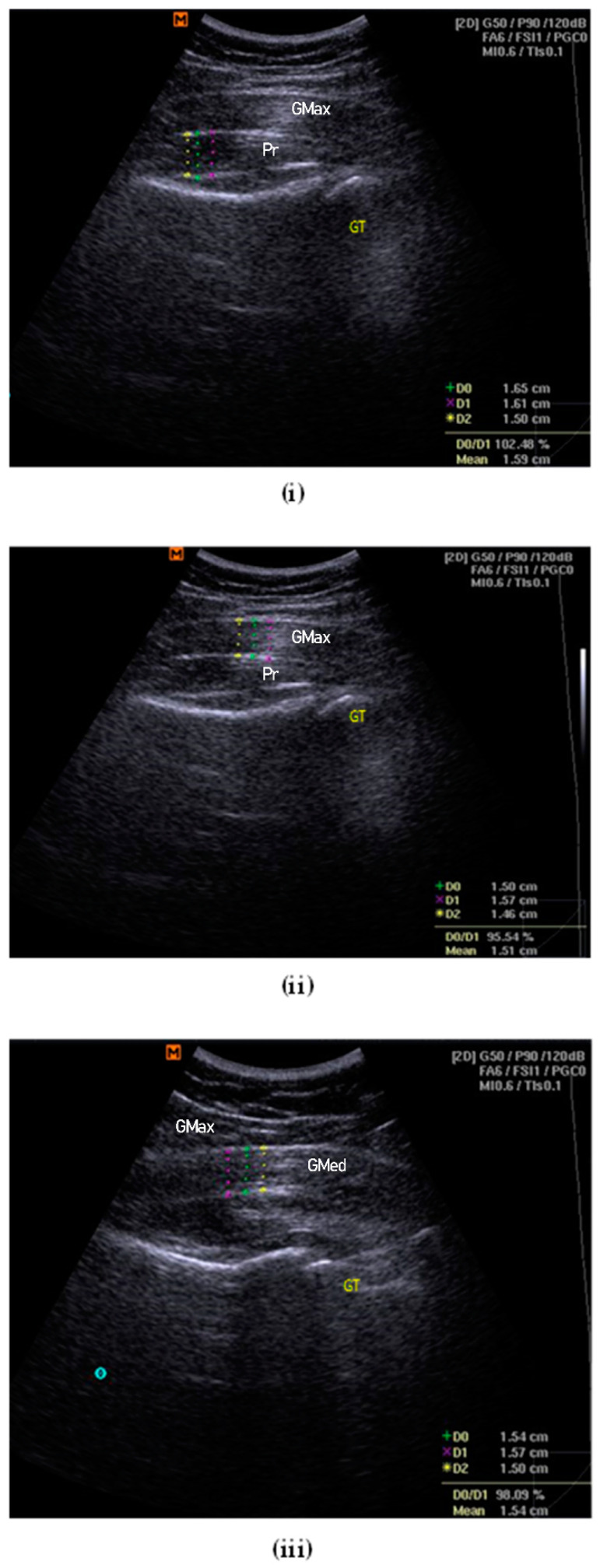
Examples of thickness measurement for piriformis (**i**), gluteus maximus (**ii**) and gluteus medius (**iii**). GT = greater trochanter of femur.

The participant was asked to perform an isometric contraction into a hip lateral rotation for several trials to assist with and confirm the identification of the piriformis muscle and fascial structures on the diagnostic USG viewscreen. The femoral greater trochanter was referred to as the main reference point, combined with the visual gliding of the piriformis muscle and the associated fasciae [25]. The depth and focus of the USG image were adjusted to portray the best possible image on the viewscreen. The thickness at the centre of the image was measured, considering the largest portion of muscle mass and excluding the surrounding fasciae that border the muscles [28]. The mean of three measurements of thickness was used for statistical analysis [28] (Figure 1i).

#### 2.7.5. Gluteus Maximus Muscle Thickness

The participant was instructed to lie down in the prone position with both lower limbs at rest and in a neutral position. A curvilinear transducer was set at a frequency of 2.0–5.5 MHz to collect B-mode ultrasound images. During image acquisition, the transducer head was coated with a generous amount of ultrasound water-based transmission gel. The transducer was placed perpendicular to the skin and the lightest contact pressure was applied to ensure that the underlying tissues were not compressed. Measurement was taken at the first third, proximally 30%, between the PSIS and the greater trochanter of the femur [28]. The depth and zoom-in function were adjusted until the image of the sacrum could not be seen in the left third [29]. Once the best possible image was shown on the viewscreen, the image was frozen and the thickness of the gluteus maximus fibre was measured. The mean of the three measurements of thickness was used for statistical analysis [28] (Figure 1ii).

#### 2.7.6. Gluteus Medius Muscle Thickness

The participant was then instructed to lie on their side with the test leg up. The tested hip was placed in a neutral flexion/extension, neutral rotation, and at 20° of adduction (inclinometer confirmed) [30]. The tested knee was in full extension, with a Biofeedback^®^ placed under the ankle and foot to ensure the resting position. A curvilinear transducer was set at 5.0 MHz (40-mm footprint; lateral and axial resolution of 1.0 and 0.93 mm, respectively) to collect bilateral anonymised resting B-mode ultrasound images [30]. Again, during image acquisition, the transducer head was coated with ultrasound water-based transmission gel and placed perpendicular to the skin. The lightest contact pressure was applied to ensure that the underlying tissues were not compressed. The transducer was placed midway between the proximal end of the iliac crest and the greater trochanter, approximately on the lower half of a coronal line located between the top of the greater trochanter and a point 25% of the distance between the ASIS and PSIS [30,31]. The cranial-caudal position of the transducer was adjusted until the superior lip of the acetabulum was one-third of the distance from the right border of the image [30]. The depth and zoom function were adjusted to obtain the best possible image before freezing. The thickness of the gluteus medius muscle was measured using callipers and it was considered as the distance between the inside edges of the muscle border, excluding the perimuscular fasciae. The mean of the three measurements of thickness was used for statistical analysis [28] (Figure 1iii).

#### 2.7.7. Measurement of Muscle Activation Using an Electromyogram (EMG)

A surface electromyogram (sEMG), a MyoTrac^®^ Biograph Infiniti System (SA7525, Thought Technology Ltd., New York, NY, USA), was employed to assess muscle function through neuromuscular electrophysiology activity [32]. Prior to the test, the hairy buttock areas of participants’ dominant legs were shaved for electrode placement. The skin was swabbed with an alcohol swab to prevent any surface interference and impedance. The electrode placement was derived from the Surface Electrode Non-Invasive for Assessment of Muscle (SENIAM) guidelines and the sensor was secured with masking tape to prevent loose contact with the targeted skin [33]. As the sEMG system was non-wireless, the wires were secured against the body to prevent pulls on the sensors and placed in a way that allowed the patient’s movement patterns to be free.

The installation of the sEMG and the software was completed before the participants’ preparation. A one-channel sEMG was used throughout the tests. Muscle electrical activity, measured during muscle contraction and relaxation cycles, was conveyed as sEMG signals in micro-Volt (μV) units. The sEMG signal processing methods were undertaken in two ways. (1) A raw or unprocessed sEMG, comprising a collection of positive and negative electrical signals. The frequency and amplitude provide information on the contraction or resting state of the muscle. (2) Root Mean Square (RMS): a technique for rectifying the raw signal and converting it into an amplitude envelope for easier viewing. It represents the mean power of the signal.

Muscle strength was measured using maximum voluntary contraction (MVC) methods for the gluteus maximus and gluteus medius, in which the muscles were activated at their maximum capabilities in the sEMG signals. Muscle activations were measured by analysing the mean RMS of the sEMG signals for each targeted muscle and task. The participants were then instructed to perform two tasks: (i) prone lying with the hip externally rotated, abducted, and slightly extended [15] and (ii) standing on a single dominant leg (SLS) [12]. Both positions were the best way to identify the activity of these muscles in incorporation actions and observe their mutual effects.

Normalisation was computed from the sEMG envelope (measured in mean RMS) of performing the task to the maximum peak value of the isometric MVC of a particular muscle (% MVC). Participants were allowed one attempt for familiarisation. Each task involved a 10-s hold for precise onset and offset sEMG signal detection. Participants attempted three repetitions with a one-minute rest in between to avoid the effects of fatigue. The same examiner (IKO) applied manual resistance during the procedures. The mean RMS values from three trials were computed automatically by the sEMG analysis software.

#### 2.7.8. Intraclass Correlation Coefficient (ICC) of the sEMG and Manual Resistance for Maximum Voluntary Contraction

Intraclass correlation coefficients (ICC) for the sEMG were obtained before actual data collection. The same procedures applied for the ultrasonography measurement were employed, comprising a pilot study, intrarater reliability analysis, and estimation of ICC values. Resultantly, the ICC yielded excellent reliability for the sEMG measurement in terms of muscle strength and activation (ICC = 0.97, 95% CI 0.91–0.99).

Manual resistance to maximum voluntary contraction (MVC) was conducted based on the interrater reliability approach to manual muscle resistance interrater reliability. Three raters were selected from among a group of experienced physiotherapists of different genders, including the main examiner (IKO). Based on a single measurement, absolute agreement, and two-way random effects, the ICC was estimated to have a moderate degree of reliability in terms of manual resistance to MVC of the gluteus maximus and gluteus medius muscles (ICC = 0.63, 95% CI −0.16–0.90).

#### 2.7.9. Muscle Strength

The gluteus maximus muscle strength was measured with the participants lying prone on the examination table. The 10 mm × 20 mm sensors were placed at 34% of the distance from the second sacral vertebrae to the greater trochanter, starting from the second sacral. The distance between the two sensors was kept to a 20 mm width and directed along the line from the posterior superior iliac spine to the middle of the posterior aspect of the thigh. A reference sensor was placed at the first lumbar spine (L1). Each participant was asked to extend the tested hip to maximum contraction against the main examiner’s (IKO) resistance.

For the gluteus medius, participants were instructed to lay on their side on the examination table with the tested leg up while the non-tested leg was positioned with the hip and knee flexed at 90. A sensor was sited at a 33% distance from the iliac crest to the greater trochanter, starting from the greater trochanter [34]. A reference sensor was placed at the first lumbar spine (L1). The tested legs were spread to maximum contraction against the manual resistance of the main examiner (IKO), who held the participant at the ankles.

#### 2.7.10. Muscle Activation

With the same sensor placement tailored for the gluteus maximus, the participants were asked to stand on the tested leg while flexing the untested leg at 900 hip and knee flexion. Participants were required to hold in the SLS position for 10 s. Activation was measured based on the onset and offset sEMG amplitude of the gluteus maximus while sustaining the SLS position. For security, a chair was made ready by the side of the participant in case they experienced instability during the task. The gluteus maximus muscle activation was measured while the participant laid prone with the tested hip externally rotated and slightly abducted and extended (ERABEX). Participants were required to hold the tested hip in the ERABEX position for 10 s. Activation was measured based on the onset and offset sEMG amplitude of the gluteus maximus while maintaining this position. A similar procedure was employed in measuring the muscle activation for the gluteus medius. For security, a chair was placed by the side of the participant in case they experienced instability during the task. Participants were required to externally rotate, slightly abduct, and extend the tested leg and hold this for 10 s. The pattern of sEMG amplitude of the gluteus medius while holding this position was measured for analysis.

### 2.8. Data Analysis

Data were analysed using SPSS version 26. Descriptive analyses were computed for the participants’ anthropometric and sociodemographic characteristics, as well as physical examinations and parameters relating to exposure to physical activities. The Shapiro-Wilk normality test found significance levels greater than 0.1, providing no evidence that the individual group data were not normally distributed. Continuous data were documented in mean (M) and standard deviation (SD) for the continuous data, whereas frequencies and percentages were presented for categorical data. Comparisons of the aforementioned data were performed using independent T-tests and Chi-square tests. Meanwhile, a one-way analysis of variance (ANOVA) was employed in comparing the muscle thickness, strength, and activation between the three groups. Assumptions of, normality, and homogeneity of variances of the residuals were met. A whisker box plot and Levene’s test were applied to assess the distribution and variance of the numerical data. A post-hoc test was conducted for the indicated variables. The homogeneity of covariance matrices and variances among the dependent variables (DV) across the groups were equal (Box’s M value and Levene’s test *p* > 0.05).

For correlation analysis, the Pearson (*r*) correlation was used to measure the degree of the relationship between the linearly related variables (piriformis thickness and hip muscle thickness, strength, and activation) in the LBP + PS and LBP − PS groups. Factors associated with piriformis thickness were identified using stepwise multiple linear regression analysis. Preliminary analyses were performed to ensure there was no violation of the assumption of normality and linearity. Linear regression was used to measure the regression between the gluteus maximus and gluteus medius MVC in two different positions: prone lying with the hip ERABEX and single leg standing. Lastly, age and gender were included in the regression analyses to determine their effects on the relationship between the selected variables in the regression model for LBP + PS and LBP − PS.

## 3. Results

### 3.1. Participants’ Anthropometric and Sociodemographic Characteristics

Table 1 displays the sociodemographic and anthropometric characteristics of the participants. Most of the participants in this study were females, accounting for more than 60.0% in each group. There is significant difference in age between LBP participants and healthy participants (*p <* 0.001). The LBP participants aged between 34.44 ± 8.21 and 36.72 ± 9.26 years old compared to healthy subjects (28.48 ± 9.95 years old). The anthropometric data showed significant (*p <* 0.05) differences in body weight in the LBP participants (73.84 ± 18.29 kg) compared to the healthy participants (63.95 ± 20.00 kg). LBP patients had greater BMI, body fat composition, and muscle mass (*p <* 0.05) compared to healthy participants. Nevertheless, no significant differences were detected in height and muscle mass.

Married individuals and employees comprised a significantly (*p* < 0.001) higher number of LBP + PS participants compared to their counterparts, whereas no significant differences in smoking and physical activity were found within all the groups. Muscle thickness was shown to be significantly thicker for the piriformis (*p* < 0.001), gluteus maximus (*p* < 0.001), and gluteus medius (*p* < 0.05) in LBP participants compared to the healthy group. However, only gluteus maximus muscle strength was significantly stronger in LBP compared to healthy participants (*p* < 0.05).

### 3.2. Participants’ Physical Examinations and Exposure to Physical Activities

Table 2 depicts physical examinations and exposure to physical activities among LBP + PS and LBP − PS participants. More than two-thirds of the LBP + PS recorded a previous gluteal injury following trauma, a fall, heavy lifting, prolonged driving, or sitting. The majority of PS participants presented with radiculopathy and gluteal tenderness on the ipsilateral side. Meanwhile, about half of the PS participants were palpated with a sausage-like mass in the piriformis region. Most participants demonstrated positive responses to the FAIR test and the Active Piriformis test, followed in order of positive responses by the Lasègue sign, HCLK, and SLR.

Participants’ distribution in terms of job fields of those affected by PS, number of hours spent daily in sitting and standing positions, and daily task-requiring prolonged sitting are presented in Table 2. Most participants were involved white-collar workers (30.56%); followed by those in the services sector, such as cleaners and couriers (13.89%); healthcare workers (11.11%); machine/construction workers and sales workers (8.33% each); and agricultural workers (2.78%). Approximately 40% and 52% of the LBP + PS participants sat and stood for four to six hours daily, respectively, compared to non-PS and healthy participants, but the difference was not statistically significant. Significant differences were detected across groups in terms of daily tasks requiring prolonged sitting. Among the LBP + PS participants, task-required occupational sitting showed the greatest proportion compared to the other activities, whereas no statistical significance was found in task-required prolonged standing.

### 3.3. Between-Group Comparisons of Piriformis and Gluteus Muscles Thickness

Piriformis thickness was significantly different (*p* < 0.05) between the groups (F (2, 83) = 5.03, *p* < 0.001). A post-hoc comparison using the Bonferroni test indicated that the mean thickness of LPB + PS was significantly (*p* < 0.01) greater, by 0.5 cm, compared to the healthy group (Table 3). Gluteus maximus thickness also showed an increasing trend among LBP + PS (2.62 ± 0.54 cm) and LBP − PS (2.52 ± 0.69 cm) participants compared to healthy participants (2.52 ± 0.69 cm). There was a significant effect in all the groups on gluteus maximus thickness (F (2, 83) = 3.96, *p* = 0.02). The Bonferroni post hoc test indicated a significant (*p* < 0.05) effect between the LBP + PS and healthy groups (Mean Difference; MD = 0.44, SD = 0.17, 95% CI: 0.03–0.86). As for the gluteus medius thickness, there was no significant (*p >* 0.05) difference between all the groups (F (2, 83) = 2.79, *p* = 0.06) Table 3i,ii.

### 3.4. Comparisons of Gluteus Muscles Strength and Activation

The LBP − PS group demonstrated the strongest gluteus maximus strength (72.59 ± 27.12 μV) compared to the other two groups (Table 4). The muscle activation of the gluteus maximus and gluteus medius measured while prone lying with the hip ERABEX (gluteus maximus F (4, 48) = 1.29, *p* = 0.28 vs. gluteus medius F (2, 48) = 0.64, *p* = 0.52) and SLS (gluteus maximus F (4, 92) = 0.41, *p* value = 0.66, gluteus medius F (2, 48) = 0.95, *p* = 0.39) was not statistically different between the three groups. However, the Bonferroni post hoc test showed a significant difference between the LBP + PS and LBP − PS groups (MD = 21.90, SD = 8.81, 95% CI: 0.04–43.75) (Table 4i,ii).

### 3.5. Correlation between Piriformis Thickness and Gluteus Muscle Strength

**Figure 2:**
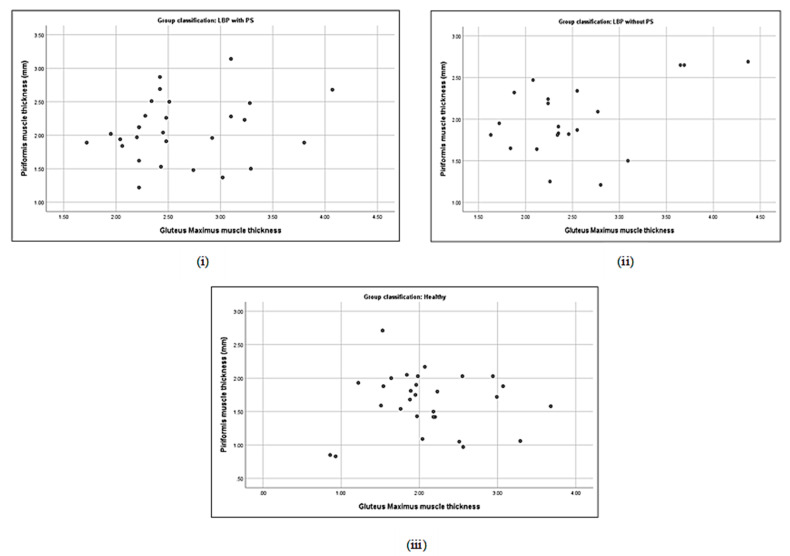
Correlations between piriformis thickness and gluteus maximus thickness in LBP + PS (**i**), LBP − PS (**ii**), and healthy (**iii**).

**Figure 3:**
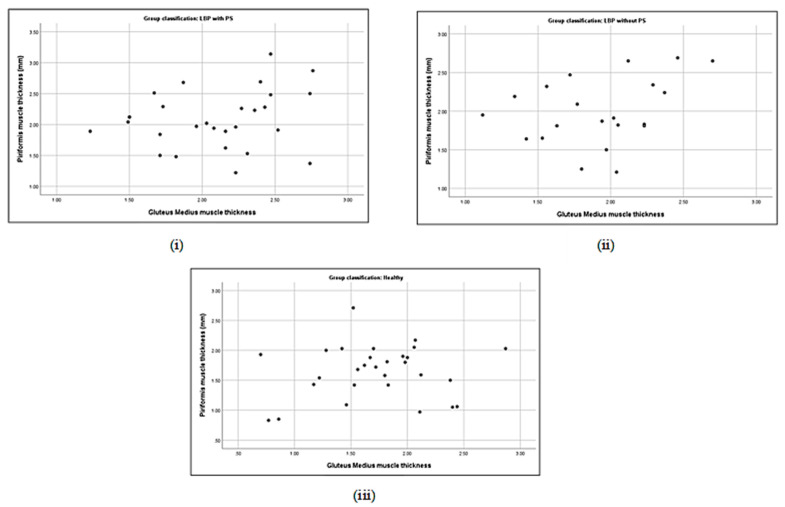
Correlations between piriformis thickness and gluteus medius thickness in LBP + PS (**i**), LBP − PS (**ii**), and healthy (**iii**).

**Figure 4:**
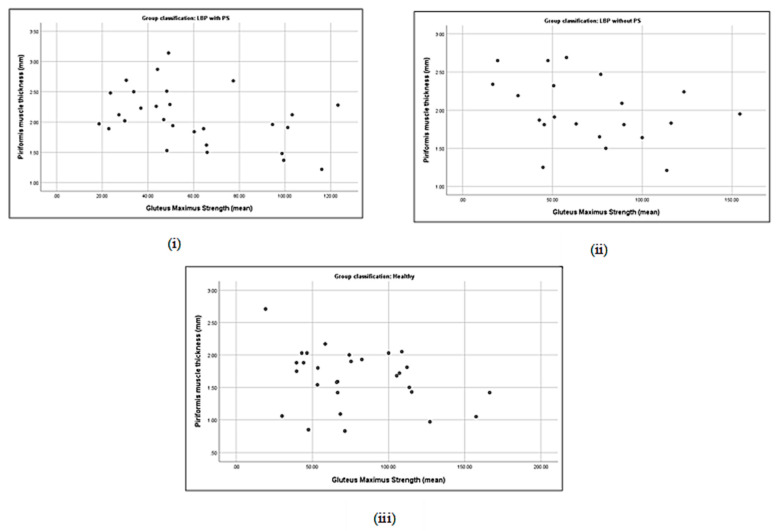
Correlations between piriformis thickness and gluteus maximus strength in LBP + PS (**i**), LBP − PS (**ii**), and healthy (**iii**).

**Figure 5:**
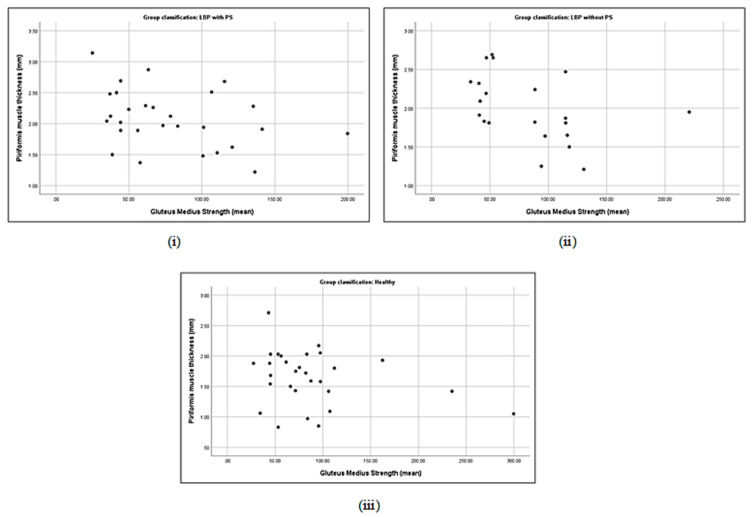
Correlations between piriformis thickness and gluteus medius strength in LBP + PS (**i**), LBP − PS (**ii**), and healthy (**iii**).

**Figure 6:**
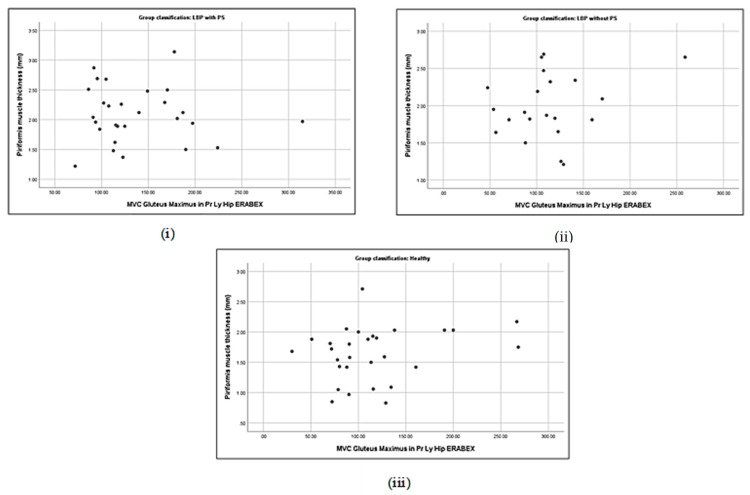
Correlations between piriformis thickness and gluteus maximus activation in prone lying with hip ERABEX in LBP + PS (**i**), LBP − PS (**ii**), and healthy (**iii**).

**Figure 7:**
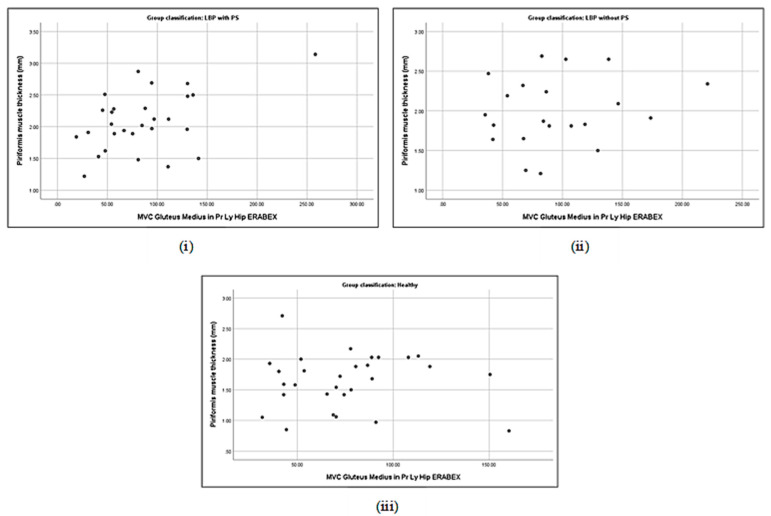
Correlations between piriformis thickness and gluteus medius activation in prone lying with hip ERABEX in LBP + PS (**i**), LBP − PS (**ii**), and healthy (**iii**).

**Figure 8:**
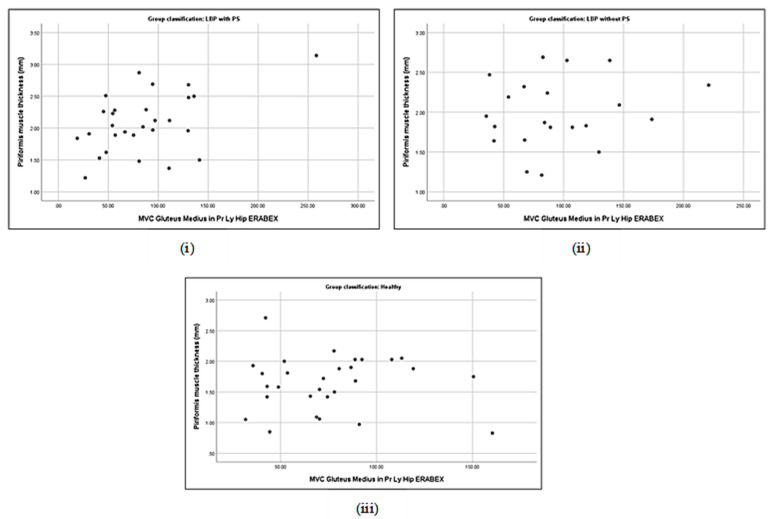
Correlations between piriformis thickness and gluteus maximus activation in single leg standing in LBP + PS (**i**), LBP − PS (**ii**), and healthy (**iii**).

**Figure 9:**
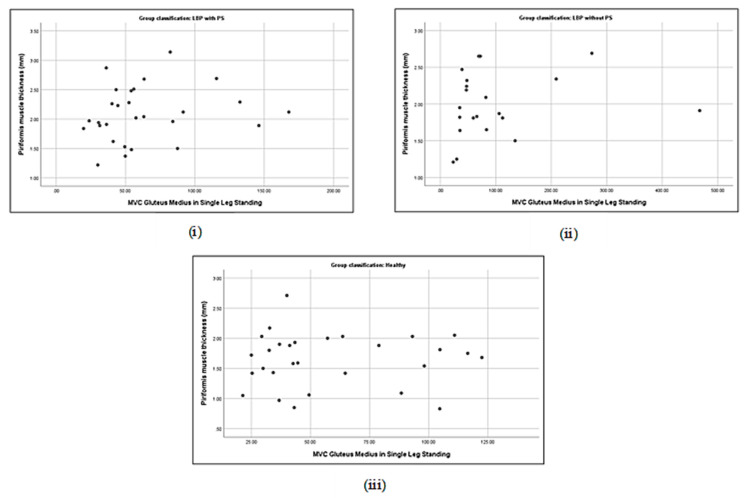
Correlations between piriformis thickness and gluteus medius activation in single leg standing in LBP + PS (**i**), LBP − PS (**ii**), and healthy (**iii**).

No significant correlation was detected either between piriformis thickness and gluteus maximus (*p* = 0.19) or gluteus medius thickness (*p* = 0.21) in the LBP + PS group (Table 5). Piriformis thickness was significantly correlated with gluteus maximus strength (*r* = −0.4, *p* < 0.05) and gluteus medius prone lying with the hip ERABEX (*r* = 0.48, *p* < 0.01). Specifically, piriformis thickness was correlated with a 40% decline in gluteus maximus strength and a 48% increase in gluteus medius activation when lying prone with the hip ERABEX. Nevertheless, a positive correlation was observed between piriformis thickness and gluteus maximus muscle thickness (*r* = 0.44, *p* < 0.05) and a negative correlation with gluteus medius strength (*r* = −0.43, *p* < 0.05) in the LBP − PS group (Table 5 and Figure 2, Figure 3, Figure 4, Figure 5, Figure 6, Figure 7, Figure 8 and Figure 9).

### 3.6. Association between Piriformis Thickness and Gluteus Thickness, Strength, and Activation

A significant correlation was observed between piriformis thickness and gluteus maximus thickness for the LBP + PS participants (Table 6). The stepwise linear regression analysis revealed a significant association between piriformis thickness and gluteus maximus strength and gluteus medius activation when lying prone with the hip in the ERABEX position (F (3, 24) = 7.98, *p* < 0.001). Gluteus medius activation when lying prone with the hip ERABEX accounted for 23% of the variance, whereas gluteus medius activation combined with gluteus maximus strength accounted for 34% of the variance. Hence, gluteus maximus strength contributed only 11% of the variance. The standardised β value indicated a moderate degree of gluteus medius activation when prone lying with the hip ERABEX (0.43). Comparatively, a lower degree of importance was observed for gluteus maximus strength (−0.34) in the model.

The multiple regression analysis indicated a significant relationship between piriformis thickness, gluteus maximus strength, and gluteus medius activation when lying prone with the hip ERABEX, after adjusting for age and gender (F (4, 23) = 3.14, *p* < 0.05). In other words, 35% of the variation in the relationship was explained by age and gender (*R*^2^ = 0.35) but no independent effect of both covariates was found within the examined range (Table 7). Thereafter, gluteal maximum no longer demonstrated a significant association with piriformis thickness, but gluteus medius activation when lying prone with the hip ERABEX was still significantly associated with piriformis thickness.

For the LBP − PS group, only gluteus maximus thickness was significantly associated with piriformis thickness, hence the variable was selected. The regression model (F (1, 19) = 4.67, *p* < 0.05) indicated that 19% of the variation in piriformis thickness was explained by gluteus maximus thickness with a corresponding moderate degree of standardised *β* value (0.44). Furthermore, the model indicated no multicollinearity problem based on the tolerance and VIF values. The shrinkage of the adjusted *R*^2^ from the actual *R*^2^ was small, denoting that the number of variables in the model was not excessive and that the sample size was sufficient. The regression model of the relationship between piriformis thickness and gluteus maximus thickness was stratified, with age and gender as potential covariates. After adjusting for age and gender, no significant association was detected between piriformis thickness and gluteus maximus thickness (F (3, 17) = 2.07, *p* > 0.05). Additionally, there were also no independent effects of age and gender within the range (Table 8).

## 4. Discussion

This study offers a new dimension of information regarding the muscle actions and morphology alteration within the lumbopelvic region. The findings of the study revealed that the piriformis muscle was thicker in LBP + PS patients compared to LBP − PS and healthy groups. This result resembles the reports by Wu et al. (2020) [35], in which a significant increase in piriformis thickness was associated with an increase in sciatic nerve (SN) pain and a decrease in the echo intensity of symptomatic legs among PS participants compared to the healthy group. Several case studies have also discovered an increase in piriformis thickness and the cross-sectional area as measured by USG imaging in chronic PS adults [17,27]. A common affirmation of the clinical diagnosis of PS is the significant increase in piriformis muscle thickness within 3 Hz to 5 Hz of USG imaging [27,35]. Hypertrophy of the piriformis has been attributed to adhesion, haematoma, spasm, and inflammation [36].

In this study, the piriformis thickness might have been caused by previous gluteal injury and microtrauma following overuse and persistent strain during prolonged occupational sitting. Previous research has highlighted a higher prevalence of piriformis overuse or injury at 19.3% [17] and 47.25% [10] compared to other traumatic injuries. A study of elite football players reported that piriformis overuse following repetitive motor control training caused an increase in muscle size, higher incidence of lower limb injuries, and LBP [37]. Piriformis overuse also induced microtrauma through persistent mechanical stress to the muscle resulting from long-distance walking or running, or by direct compression due to repetitive trauma from sitting on hard surfaces. As observed in this study, 59.9% of the LBP + PS participants experienced long hours of sitting (>4 h/day) and 60% were involved in occupational sitting at their workplace. Long-term microtrauma from overuse injuries can lead to scarring of the piriformis fibre [9], muscle hypertrophy, and PS. However, piriformis thickness was not statistically different between the LBP + PS and LBP − PS groups. This might be attributed to the increase in persistent piriformis tension in both patients as any instability in the trunk, such as a lack of motor control of the deep trunk muscle, multifidus, or transverse abdominalis [38], would result in the active exhibition of the lumbopelvic-hip musculature, including piriformis, to make the imbalance subside.

Gluteus maximus thickness was found to be significantly different across the groups. The LBP + PS participants differed significantly in gluteus maximus thickness by 0.5 cm relative to healthy adults. Meanwhile, an increase in thickness by 0.1 cm in the LBP − PS group was not statistically different compared to the healthy participants. This finding indicated that the gluteus maximus becomes hypertrophic in chronic LBP and even slightly more so in PS patients. Previous studies have produced inconsistent findings on the gluteus maximus muscle size in LBP patients and healthy individuals [39,40]. Nonetheless, the present result aligns with studies reporting positive associations between LBP and trunk muscle weakness [38,41]. A recent study revealed that core stability training addressed the significant thickness of the contracted gluteus maximus and reduced physical disability among NSLBP individuals [42].

In the current study, LBP with PS individuals may have higher compensatory gluteus maximus activation to counterbalance the piriformis inactivation. Partly, irritation to the gluteal vessel contributed to micro haematoma and also executed inflammation in the gluteal muscles, thereby increasing the gluteus muscle diameter. In line with the present study, the results revealed that 77.8% of participants in the LBP + PS group had a previous gluteal injury and presented with gluteal tenderness (100%). Such an event was lacking in the LBP − PS group, as there were fewer constraints at the gluteus but, rather, at the lower back muscles [42]. Thus, an increase in gluteus maximus muscle recruitment resulted in greater increases in muscle mass and fibre size. Meanwhile, the insignificant results in the gluteus medius thickness between the various groups contradict those of previous studies [43,44,45].

Variations were observed in the strength and direction of the correlation between piriformis muscle thickness on one hand and gluteus maximus/gluteus medius muscle thickness, strength, and activation on the other. Piriformis thickness was negatively and moderately correlated with gluteus maximus strength (*r* = −0.4) and positively correlated with gluteus medius activation (*r* = 0.48) in the LBP + PS group. Comparatively, LBP − PS depicted a positive and moderate correlation with gluteus maximus strength (*r* = 0.44) and an inverse correlation with gluteus medius strength (*r* = −0.43). These findings align with the conclusion drawn from a systematic review of high-quality studies (*n* = 674), which demonstrated a significant decline in the CSA and muscle volume (3D) of the hip muscles (i.e., gluteus maximus, gluteus medius, gluteus minimus, and piriformis) in LBP patients [45].

The inverse relationship between gluteus maximus strength and the piriformis might be linked to findings reported in earlier research. PS was mostly recognised in an overused piriformis due to a prolonged sitting position with the hip externally rotated and flexed [7]. This position caused a notable increase in the synergistic activation of the piriformis in maintaining the lumbopelvic-hip static posture. Prolonged sitting resulted in gluteus maximus deformation and increased fatty tissue composition, eventually reducing the muscle volume [46]. Hence, gluteus maximus strength diminished even more in PS patients as observed in the present study. Several reports have depicted a decline in gluteus muscle morphology in the presence of LBP [45,47]. However, most of the evidence involved discogenic LBP and entrapment neuropathies of the hip. Studies on non-discogenic conditions such as deep gluteal pain, including PS, and their relationship with gluteal muscle activities remain scarce and conflicting. Further studies are warranted to explore this relationship in detail. This study observed no influence of the gluteus medius in predicting piriformis thickness among the LBP + PS group, but selected cases were found in the LBP − PS group, which coincides with earlier reports [44,45]. No studies have discussed gluteus medius morphological changes, particularly in PS, making it difficult to identify the association between them.

In the regression analysis, gluteus maximus thickness predicted piriformis thickness in the LBP − PS group. This finding indicated that the local and global hip muscles were elicited simultaneously to counteract the subsequent perturbations of trunk equilibrium. Neumann (2010) [12] discovered the biomechanical interaction between the primary hip external rotator, gluteus maximus, piriformis, and other short external rotators, including the gluteus medius. An increase in biomechanical compensatory workload on the spine results from activities such as lower limb prolonged walking or standing on a single leg with poor trunk control. These events trigger excessive pressure on the lumbopelvic-hip region, eventually resulting in excessive coactivation of the local hip muscles, including the piriformis. Hence, aligning with the present findings, an increase in coactivated gluteus muscle mass is related to an increase in piriformis thickness in the presence of LBP.

The association between LBP and gluteus muscle activation has been described in several studies with inconsistent results [34,48,49]. Only a few studies have been conducted on piriformis activation due to its deep location in the greater sciatic notch [15]. In the current study, the simple regression analysis revealed that the gluteus maximus and gluteus medius muscle activation were not associated with piriformis thickness. This finding contradicts a previous study that demonstrated a strong association between all the quadriceps muscles’ thickness and muscle activation, measured by EMG amplitude distribution [50]. Variations in studies regarding the measurement of changes in piriformis thickness during maximum contraction might explain these discrepancies [51,52].

The RMS amplitude distribution was also demonstrated to be uneven in the isometric contraction while in the SLS position and prone lying with the hip externally rotated, slight abducted, and extended. Despite these positions being ideal for possible integrating the movement of piriformis-gluteus activations [12,15], no associations in any of the groups were observed in the present study. Evidence from previous research highlighted the influence of postural sway and amplitude inconsistency during sEMG [53]. While measuring the sEMG in our study, the piriformis, gluteus maximus, and gluteus medius muscles might have greater perturbations of anticipatory activations during single leg standing compared to double leg standing. Besides, the muscle capability to restrain postural stability increased when held for some periods and with some repetitions in several trials. Therefore, measurements of the genuine RMS amplitude of the piriformis-gluteus activation were difficult to obtain. Likewise, prone lying with the hip ERABEX was challenging for individuals with active pain levels. In the LBP + PS group, due to gluteal tenderness, the rough RMS amplitude was observed while performing the task of prone lying with the hip externally rotated, abducted, and extended. This could explain the insignificant association between these variables.

Age was not well distributed as a participant characteristic across all the groups. Previous studies firmly agree that increasing age significantly increases the risk of LBP. In this study, the mean age group was 35.96 ± 8.68 years old, classified as the productive age group or working-age adults. Working-age adults in the age group 30 to 49 [35] are critically predisposed to mechanical LBP due to occupational hazards [54]. However, our findings could not identify the effect of ageing on the relationship between piriformis and gluteus muscle thickness, strength, and activation. This suggests that the age factor could not be solely distinguished as confounding in the regression between piriformis and gluteus thickness, strength, and activation, but it may mediate the effect of gluteus maximum strength on piriformis muscle thickness.

Overall, women contributed a larger proportion of LBP + PS (60%) compared to men. The higher risk of LBP among females has been linked to gender variations in anatomical architecture [55]. Similarly, the incidence of LBP in PS patients was more common in women than men, with a 6:1 ratio [56]. Upon adjusting for gender, gender did not interact in the regression model of the relationship between piriformis and gluteus thickness, strength, and activation. No evidence can be found in the literature concerning this finding. Nevertheless, the relationship between gender and muscle function or muscle morphological changes has been investigated separately in the available literature. Miller et al. (2021) [57] found that knee extensor force significantly declined with age among women of various age groups. Another study reported that gluteus maximus showed a significantly smaller CSA in women with chronic LBP compared to healthy women. Meanwhile, research on the piriformis muscle and its relationship with gender is also limited. Mondal et al. (2017) [58] found that significant tightness of the piriformis muscle was similar in both males and females but for different reasons. The causes of tightness in males are mostly related to heavy lifting and work-related injury, while for women, sitting cross-legged during house chore activities significantly increased the tension of the piriformis muscle. This study only observed the differences between the genders but did not measure the interactions of the adjustments for gender in the regression. Examining the existing evidence, the effect of gender may interact separately with the parameter, but this was not reflected in the regression analysis. Conclusively, gender does not independently influence the relationship between piriformis and gluteus muscle thickness, strength, and activation.

The limitations in this study are well-acknowledged. Given that piriformis muscle activity was not measured, its relationship with gluteus muscle activity could not be investigated. The reason for this weakness is the complexity of measuring the H-reflex in piriformis muscle activities using a fine-needle EMG. Additionally, the various causes of thickness were not distinguished, with muscle thickness possibly observed as adhesion, mass, spasm, or inflammation in the muscle fibre properties. Analyses of the muscle composition through muscle echogenicity quantification were not performed, which could have defined and classified the causes of thickness. The results should be interpreted with caution, given the unmatched number and age distribution of participants in each group and since sEMG of the gluteus medius muscle does not reflect only an activation of the g.medius muscle only. It may include an activation of the maximus muscle because it is an inner muscle and located under the maximus muscle. Lastly, non- discogenic pain was distinguished through negative radiology imaging, although magnetic resonance imaging is more accurate and reliable in determining the involvement of intervertebral disc disorders.

## 5. Conclusions

This study found that piriformis thickness is inversely associated with gluteus maximus strength but positively associated with gluteus medius activation. Thus, those with PS might recruit more actions in the gluteus medius compared to the gluteus maximus for static or dynamic stability. This information might assist clinicians to manage PS more effectively and precisely, as well as resolving the discrepancy in the piriformis-gluteus interactions. Information on this relationship is important in both clinical practice and research as it elucidates the interactions between those muscles in terms of actions and elements.

## Figures and Tables

**Table 1:** Baseline characteristics among low back pain and healthy participants.

Variables	LBP + PS (*n* = 36; 40%)	LBP − PS (*n* = 25; 27%)	Healthy (*n* = 30; 33%)	*p*-Value
**Gender**				0.79
Male	12 (41.38)	8 (27.59)	9 (31.03)	
Female	24 (38.72)	17 (27.42)	21 (33.87)	
**Age, years**	36.72 ± 9.26	34.44 ± 8.21	28.48 ± 9.95	<0.001 ***
**Height, m**	1.61 ± 0.09	1.59 ± 0.08	1.58 ± 0.07	0.59
**Weight, kg**	74.35 ± 17.38	72.34 ± 19.44	63.95 ± 20.00	0.02 *
**BMI, kg/m^2^**	28.83 ± 6.76	28.27 ± 6.23	25.09 ± 7.06	0.01 **
**Body fat, %**	37.65 ± 11.72	37.20 ± 9.80	32.45 ± 8.29	0.02 *
**Muscle mass, %**	29.06 ± 7.37	26.92 ± 6.08	25.72 ± 5.86	0.09
**Muscle thickness, cm**				
Piriformis	2.09 ± 0.50	1.99 ± 0.43	1.64 ± 0.44	<0.001 ***
Gluteus maximus	2.62 ± 0.54	2.52 ± 0.69	2.10 ± 0.66	<0.001 ***
Gluteus medius	2.08 ± 0.43	1.92 ± 0.39	1.73 ± 0.51	0.02 *
**Muscle strength, μV**				
Gluteus maximus	50.69 ± 29.89	72.59 ± 27.12	59.27 ± 18.52	0.04 *
Gluteus medius	73.44 ± 32.93	82.64 ± 32.98	73.27 ± 34.84	0.53
**Smoking**	6 (85.71)	1 (14.29)		0.19
Yes	30 (54.56)	24 (43.67)	1 (1.82)	
No	2 (5.71)	5 (14.29)	28 (80.00)	
**Physical level**				0.27
Active (>2 strenuous/week)	34 (57.63)	20 (33.90)	5 (8.47)	
Inactive (<2 strenuous/week)	2 (6.45)	5 (16.13)	24 (77.41)	
**Marital status**	24 (58.54)	17 (41.46)		<0.001 ***
Married	12 (41.38)	8 (27.59)	9 (31.03)	
Unmarried			20 (100.00)	
**Employment status**	27 (60.00)	18 (40.00)		<0.001 ***
Employed	4 (28.57)	3 (21.43)	7 (50.00)	
Unemployed	4 (14.81)	1 (3.70)	22 (81.48)	

BMI = body mass index; LBP = low back pain; * *p* < 0.05, ** *p* < 0.01, *** *p* < 0.001.

**Table 2:** Physical assessment and exposure related to piriformis syndrome.

Variables	LBP + PS (*n* = 36; 40%)	LBP − PS (*n* = 25; 27%)	Healthy (*n* = 30; 33%)	*p*-Value
**Radiculopathy**				
Yes	25 (69.40)	7 (28.00)	0	<0.001 ***
No	11 (30.60)	18 (72.00)	30 (97.00)	
**Current history: Trauma/Fall/Heavy lifting**	28 (77.80)	13 (52.00)	2 (6.70)	<0.001 ***
Prolonged driving/sitting	2 (5.60)	1 (4.00)	0	<0.001 ***
Presence of gluteal tenderness	36 (100.00)	15 (60.00)	6 (20.00)	<0.001 ***
Presence of a sausage-shaped mass	15 (41.70)	1 (4.00)	0	<0.001 ***
**Positive special test**				<0.001 ***
SLR	21 (58.30)	3 (12.00)	0	
HCLK	24 (66.70)	1 (4.00)	0	
Active Piriformis test	34 (94.40)	12 (48.00)	2 (6.70)	
Laseuge sign	28 (77.80)	3 (12.00)	2 (6.70)	
FAIR	36 (100.00)	5 (20.00)	2 (6.70)	
**Job field**				<0.01 **
White collar	11 (45.80)	9 (37.50)	4 (16.70)	
Agriculture/Forestry	1 (2.78)	0	0	
Transportation	0	1 (100.00)	0	
Metal/Machine/Construction	3 (60.00)	2 (40.00)	0	
Clinician/Nurse/Healthcare	4 (44.40)	4 (44.40)	1 (11.10)	
Sales work/Entrepreneurship	3 (60.00)	1 (20.00)	1 (20.00)	
Services or other occupations	5 (50.00)	4 (40.00)	1 (10.00)	
**Sitting duration in a day**				0.66
<4 h	8 (32.00)	9 (36.00)	8 (32.00)	
4–6 h	23 (46.00)	11 (22.00)	16 (32.00)	
>6 h	5 (13.89)	5 (20.00)	5 (17.24)	
**Standing duration in a day**				0.41
<4 h	10 (27.78)	12 (36.40)	11 (33.30)	
4–6 h	13 (36.11)	7 (21.90)	12 (37.50)	
>6 h	13 (36.11)	6 (24.00)	6 (24.00)	
**Tasks required prolonged sitting**				<0.01 **
Occupational sitting	18 (60.00)	8 (27.70)	4 (13.30)	
Religious activity	1 (50.00)	0	1 (50.00)	
Social media (i.e.,: games, Facebook, Whatsapp)	4 (28.6)	3 (21.40)	7 (50.00)	
Leisure (i.e.,: reading/watching television)	4 (14.80)	7 (25.90)	16 (59.30)	
House chores	9 (56.30)	6 (37.50)	1 (6.30)	
**Task required prolonged standing**				0.29
Occupational standing	21 (43.80)	14 (29.20)	13 (27.10)	
Religious activity	1 (50.00)	1 (50.00)	0	
Sport activities	0	3 (42.90)	4 (57.10)	
Leisure (farming/gardening/sports)	1 (25.00)	0	3 (75.00)	
House chores	12 (42.90)	7 (25.00)	9 (32.10)	

PS = piriformis syndrome; HCLK = heel contra lateral knee; FAIR = flexion abduction internal rotation, WA = WhatsApp, ** *p* < 0.01, *** *p* < 0.001.

**Table 3:** Comparison between piriformis and gluteus muscle thickness (**i**). Pair-wise comparison of piriformis and gluteus muscle thickness between three groups (**ii**).

(**i**)									
**Thickness (cm)**	**LBP + PS** **Mean ± SD**	**LBP-PS Mean ± SD**	**Healthy Mean ± SD**	***p*-Value**	**Sum of Squares**	**df**	**Mean Square**	**F**	**Partial ETA** **Squared**
Piriformis	2.09 ± 0.50	1.99 ± 0.43	1.64 ± 0.44	*p* < 0.01	2.65	2	1.33	5.03	0.11
Gluteus maximus	2.62 ± 0.54	2.52 ± 0.69	2.10 ± 0.66	0.02 *	3.64	2	1.82	3.96	0.09
Gluteus medius	2.08 ± 0.43	1.92 ± 0.39	1.73 ± 0.51	0.06	1.40	2	0.70	2.79	0.09
(**ii**)									
**Thickness (cm)**	**Group Classification**			**Std. Error**	***p*** **-Value**		**95% CI for Difference**		
							**Lower Bound**		**Upper Bound**
Piriformis	LBP − PS		Healthy	0.12	1.00		−0.18		0.38
	LBP + PS		Healthy	0.11	0.00 ***		0.08		0.61
	LBP − PS		LBP + PS	0.12	1.00		−0.39		0.19
Gluteus maximus	LBP − PS		Healthy	0.18	0.15		−0.08		0.79
	LBP + PS		Healthy	0.16	0.01 **		0.07		0.84
	LBP − PS		LBP + PS	0.17	1.00		−0.31		0.51
Gluteus medius	LBP − PS		Healthy	0.13	0.54		−0.15		0.50
	LBP + PS		Healthy	0.12	0.10		−0.05		0.62
	LBP − PS		LBP + PS	0.13	0.62		−0.15		0.46

PS = piriformis syndrome; SD = standard deviation; df = degrees of freedom; * *p* < 0.05, ** *p* < 0.01, *** *p* < 0.001.

**Table 4:** Comparison of gluteus muscle strength and activation (**i**). Pair-wise comparison of gluteus muscle strength and activation between three groups (**ii**).

(**i**)								
**Variables**	**PS** **Mean ± SD**	**Non-PS Mean ± SD**	**Healthy Mean ± SD**	***p*-Value**	**Sum of Squares**	**df**	**Mean Square**	**F**
Muscle strength (μV)								
Gluteus maximus	47.43 ± 26.54	72.59 ± 27.12	59.27 ± 18.52	0.01 *	5441.96	2	2720.98	4.53
Gluteus medius	70.29 ± 30.39	82.64 ± 32.98	73.27 ± 34.84	0.53	1364.52	2	682.26	0.64
Muscle activation prone lying with hip ERABEX (μV)								
Gluteus maximus	105.70 ± 48.64	83.35 ± 22.98	103.79 ± 51.20	0.28	4927.54	2	2463.77	1.29
Gluteus medius	65.81 ± 6.17	68.53 ± 7.30	61.17 ± 7.11	0.52	1103.12	2	551.56	0.64
Muscle activation in SLS (μV)								
Gluteus maximus	73.48 ± 64.48	63.12 ± 39.56	58.85 ± 36.21	0.66	2061.09	2	1030.54	0.40
Gluteus medius	45.30 ± 28.24	58.75 ± 34.97	56.17 ± 30.71	0.39	1837.67	2	918.83	0.95
(**ii**)								
**Variables**	**Group Classification**		**Std. Error**	***p*-Value**		**95% CI for Difference**		
						**Lower Bound**	**Upper Bound**	
Gluteus maximus strength	LBP − PS	Healthy	8.80	0.41		−8.53	35.16	
	LBP + PS	Healthy	8.21	0.46		−32.24	8.54	
	LBP − PS	LBP + PS	8.37	0.01 **		−45.93	−4.40	
Gluteus medius strength	LBP − PS	Healthy	11.71	1.00		−19.69	38.43	
	LBP + PS	Healthy	10.93	1.00		−30.09	24.14	
	LBP − PS	LBP + PS	11.13	0.81		−39.96	15.27	
Gluteus maximus activation in STS	LBP − PS	Healthy	18.03	1.00		−40.47	49.02	
	LBP + PS	Healthy	16.83	1.00		−27.12	56.39	
	LBP − PS	LBP + PS	17.14	1.00		−32.17	52.88	
Gluteus maximus activation in STS	LBP − PS	Healthy	10.62	0.63		−12.90	39.80	
	LBP + PS	Healthy	10.43	0.90		−36.75	15.00	
	LBP − PS	LBP + PS	10.62	0.63		−39.80	12.90	
Gluteus maximus activation in hip ERABEX	LBP − PS	Healthy	15.95	0.57		−60.69	18.53	
	LBP + PS	Healthy	15.07	0.44		−59.64	15.18	
	LBP − PS	LBP + PS	15.07	0.44		−15.18	59.64	
Gluteus medius activation in hip ERABEX	LBP − PS	Healthy	10.11	1.00		−17.59	32.64	
	LBP + PS	Healthy	9.48	1.00		−16.80	30.26	
	LBP − PS	LBP + PS	9.55	1.00		−24.52	22.93	

PS = piriformis syndrome; MVC = maximum voluntary contraction; ERABEX = Externally rotated, slight abducted and extended; SLS = single leg standing; SD = standard deviation; * *p* < 0.05. LBP = low back pain; CI = confidence interval; ** *p* < 0.01.

**Table 5:** Pearson correlation (*r*) between piriformis thickness, gluteus maximus muscle thickness, and gluteus medius activation among low back pain participants.

		Gluteus Maximus			Gluteus Maximus Activation	Gluteus Medius			Gluteus Medius Activation
Variables	LBP	Thickness (cm)	Strength (μV)	Prone Lying with Hip ERABEX (μV)	SLS (μV)	Thickness (cm)	Strength (μV)	Prone Lying with Hip ERABEX (μV)	SLS (μV)
Piriformis thickness (cm)	with PS	0.19	−0.40 *	−0.06	0.16	0.21	−0.33	0.48 **	0.18
	without PS	0.44 *	−0.35	0.24	0.19	0.33	−0.43 *	0.17	0.18
Gluteus maximus thickness (cm)	with PS	1.00	0.25	−0.17	−0.15	.31	−0.18	0.37 *	0.26
	without PS		−0.35	0.33	0.02	0.69 **	−0.38	0.29	0.29
Gluteus maximus activation prone lying with hip ERABE (μV)	with PS		1.00	−0.45 *	−0.55 **	0.13	0.44 *	−0.14	0.11
	without PS		1.00	0.35	−0.42	−0.34	0.64 **	−0.33	−0.32
Gluteus maximus activation in SLS (μV)	with PS			1.00	0.08	−0.07	−0.45	0.29	−0.03
	without PS			1.00	0.12	0.35	−0.30	0.31	−0.05
Gluteus maximus activation in SLS (μV)	with PS					1.00	−0.04	0.15	−0.24
	without PS					1.00	−0.51 *	0.47 *	0.32
Gluteus medius thickness (μV)	with PS						1.00	−0.58 **	−0.47 *
	without PS						1.00	−0.52 *	−0.37
Gluteus medius activation prone lying with hip ERABEX (μV)	with PS							1.00	0.31
	without PS							1.00	0.59 **

ERABEX = externally rotated, abducted and extended; LBP = low back pain; PS = piriformis syndrome; SLS = single leg standing; * *p* < 0.05, ** *p* < 0.01.

**Table 6:** Stepwise regression analyses of the associations between piriformis thickness and gluteus muscle strength and gluteus muscle activation in low back pain participants with piriformis syndrome.

	B	Β	Adjusted *R*^2^	*p*-Value	Tolerance	VIF
**Model 1**						
Constant	1.69					
MVC gluteus medius in prone lying with hip ERABEX (μV)	0.00	0.48 **	0.20	0.01	1.00	1.00
**Model 2**						
Constant	2.04					
MVC gluteus medius in prone lying with hip ERABEX (μV)	0.01	0.43 *	0.29	0.01	0.98	1.02
Gluteus maximus strength (μV)	−0.01	−0.34 *		0.04	0.98	1.02

ERABEX = externally rotated, abducted, and extended; LBP = low back pain; MVC = maximal voluntary contraction; VIF = variance inflation factor LBP with PS; *R*^2^ = 0.23 for Step 1 (∆*R*^2^ = 0.23), *R*^2^ = 0.34 for Step 2 (∆*R*^2^ = 0.11); * *p* < 0.05, ** *p* < 0.01.

**Table 7:** Regression analysis for low back pain participants with piriformis syndrome adjusted for age and gender.

Covariates	Coefficient	*p* Value	T	VIF
^a^ Gender	0.06	0.75	0.91	1.10
Age (year)	0.07	0.71	0.90	1.11
Gluteus maximus strength (mean) (μV)	−0.31	0.09	0.88	1.13
MVC gluteus medius in a prone lying position with hip ERABEX	0.43	0.02 *	0.94	1.06
^b^ Age (year)	0.08	0.66	0.92	1.09
Gluteus maximus strength (mean) (μV)	−0.33	0.07	0.93	1.07
MVC gluteus medius in a prone lying position with hip ERABEX (μV)	0.42	0.02 *	0.96	1.05
^c^ Gluteus Maximus Strength (mean) (μV)	−0.34	0.04 *	0.98	1.02
MVC gluteus medius in prone lying hip ERABEX (μV)	0.43	0.01 **	0.98	1.02

Backward multiple linear regression method. Model assumptions are fulfilled and no multicollinearity was detected. No significant interactions among independent variables. Coefficient of determination. ^a^ (*R*^2^) = 0.35; ^b^ (*R*^2^) = 0.35; ^c^ (*R*^2^) = 0.45. * *p* < 0.05, ** *p* < 0.01.

**Table 8:** Stepwise regression analyses of the association between piriformis thickness and gluteus thickness, strength, and activation in low back pain participants without piriformis syndrome, adjusted for age and gender.

Covariates	Coefficient	*p*-Value	T	VIF
^a^ Gender	−0.19	0.40	0.90	1.11
Age	0.20	0.39	0.82	1.22
Gluteus maximus muscle thickness	0.31	0.21	0.78	1.28
^b^ Age	0.22	0.35	0.83	1.21
Gluteus maximus muscle thickness	0.35	0.14	0.83	1.21
^c^ Gluteus maximus muscle thickness	0.44	0.04 *	1.00	1.00

Backward multiple linear regression method. Model assumptions are fulfilled, and no multicollinearity was detected. No significant interactions among independent variables. Coefficient of determination ^a^ (*R*^2^) = 0.27; ^b^ (*R*^2^) = 0.24; ^c^ (*R*^2^) = 0.19. * *p* < 0.05.

## Data Availability

The dataset will be provided by the corresponding author on request.
